# Efficacy of Topiramate in Treating Methamphetamine Use Disorder: A Systematic Review and Meta-Analysis of Randomized Controlled Trials

**DOI:** 10.1192/j.eurpsy.2025.969

**Published:** 2025-08-26

**Authors:** M. L. Geremias, I. Borja De Oliveira, A. L. Balduino de Souza, F. Bandeira de Melo Guimarães, D. Soler Lopes

**Affiliations:** 1 Medicine, University of Joinville Region (UNIVILLE), Joinville; 2 Medicine, University of São Paulo, São Paulo; 3 Medicine, Evangelical University of Goias, Anapolis; 4 Medicine, Federal University of Rio de Janeiro (UFRJ), Rio de Janeiro, Brazil; 5 Medicine, Lancaster University, Lancaster, United Kingdom

## Abstract

**Introduction:**

Methamphetamine use disorder poses a significant public health challenge, with few effective pharmacological treatments. Topiramate, an anticonvulsant, shows potential for treating various substance use disorders. This meta-analysis evaluates topiramate’s efficacy in treating methamphetamine use disorder, focusing on abstinence rates and depressive symptoms.

**Objectives:**

This review aims to assess the efficacy of topiramate in treating methamphetamine use disorder, specifically its impact on abstinence rates measured by negative urine tests for methamphetamine. Additionally, it evaluates topiramate’s effects on depressive symptoms, quantified by Beck Depression Inventory scores.

**Methods:**

A systematic search was conducted in Scopus, Web of Science, and PsycINFO in September 2024, following the Preferred Reporting Items for Systematic Reviews and Meta-Analyses (PRISMA) guidelines. Included studies were peer-reviewed randomized controlled trials (RCTs) assessing topiramate’s effects on individuals with methamphetamine use disorder. The analysis utilized a random-effects model, with the primary outcome being abstinence assessed through negative urine tests and the secondary outcome being depression scores from the Beck Depression Inventory.

**Results:**

Three studies (n = 249) were included, comparing topiramate to placebo. The pooled risk ratio (RR) for the common effect model was 1.00 (95% CI: 0.94-1.07), indicating no significant difference between topiramate and placebo. Heterogeneity was low (I² = 2%, p = 0.36). Individual study risk ratios ranged from 0.43 to 1.09, with the largest study (n = 140) showing no effect (RR 1.00, 95% CI: 0.93-1.07). Two studies (n = 109) reported that topiramate tended to improve depressive symptoms relative to placebo, though not reaching statistical significance (mean difference = -2.52 (95% CI: -5.31 to 0.26).

**Conclusions:**

For patients with methamphetamine use disorder, topiramate did not increase abstinence rates when compared to placebo, but showed a trend towards improving depressive symptoms. Although no statistically significant effects were observed, the trends provide a foundation for future research. Larger sample sizes, extended follow-up periods, and standardized outcome measures are needed to better evaluate topiramate’s efficacy. Future studies should also explore dose-response relationships, combination therapies, and identify patient subgroups likely to benefit from topiramate, which may reveal clinically meaningful effects and enhance treatment options for methamphetamine use disorder.

**Disclosure of Interest:**

None Declared

